# Proteomic Analysis of Lung Cancer Types—A Pilot Study

**DOI:** 10.3390/cancers14112629

**Published:** 2022-05-26

**Authors:** Simon Sugár, Fanni Bugyi, Gábor Tóth, Judit Pápay, Ilona Kovalszky, Tamás Tornóczky, László Drahos, Lilla Turiák

**Affiliations:** 1 MS Proteomics Research Group, Research Centre for Natural Sciences, Magyar Tudósok Körútja 2, H-1117 Budapest, Hungary; sugar.simon@ttk.hu (S.S.); bugyi.fanni@ttk.hu (F.B.); toth.gabor@ttk.hu (G.T.); drahos.laszlo@ttk.hu (L.D.); 2 Doctoral School of Pharmaceutical Sciences, Semmelweis University, Üllői út 26, H-1085 Budapest, Hungary; 3 Hevesy György PhD School of Chemistry, Eötvös Loránd University, Pázmány Péter sétány 1/A, H-1117 Budapest, Hungary; 4 Department of Inorganic and Analytical Chemistry, Budapest University of Technology and Economics, Szt. Gellért tér 4, H-1111 Budapest, Hungary; 5 Department of Pathology and Experimental Cancer Research, Semmelweis University, Üllői út 26, H-1085 Budapest, Hungary; papay.judit@med.semmelweis-univ.hu (J.P.); kovalszky.ilona@med.semmelweis-univ.hu (I.K.); 6 Department of Pathology, Medical School and Clinical Center, University of Pecs, Szigeti út 12, H-7624 Pecs, Hungary; tornoczki.tamas@pte.hu

**Keywords:** lung cancer, proteomics, mass spectrometry, gene set enrichment analysis, NSCLC, SCLC, cancer, human tissue, lung tissue

## Abstract

**Simple Summary:**

Tackling and curing cancer is still one of the most important challenges of biomedical research. Lung cancer is among the most diverse and lethal types, therefore identifying alterations in proteins participating in events leading to this disease is crucial. By analyzing and comparing the tissue proteomics profile of small cell lung cancer, as well as non-small cell lung cancer (adenocarcinoma, squamous cell carcinoma, large cell carcinoma) subtypes, following on-surface tryptic digestion, we aimed to identify the key dysregulated pathways. Proteins altered between cancerous and respective adjacent normal tissue were determined to reveal common and lung cancer type-specific changes. These proteins can contribute to a more precise classification of lung cancer and, following validation, can further improve the currently available diagnostic panels.

**Abstract:**

Lung cancer is the leading cause of tumor-related mortality, therefore significant effort is directed towards understanding molecular alterations occurring at the origin of the disease to improve current treatment options. The aim of our pilot-scale study was to carry out a detailed proteomic analysis of formalin-fixed paraffin-embedded tissue sections from patients with small cell or non-small cell lung cancer (adenocarcinoma, squamous cell carcinoma, and large cell carcinoma). Tissue surface digestion was performed on relatively small cancerous and tumor-adjacent normal regions and differentially expressed proteins were identified using label-free quantitative mass spectrometry and subsequent statistical analysis. Principal component analysis clearly distinguished cancerous and cancer adjacent normal samples, while the four lung cancer types investigated had distinct molecular profiles and gene set enrichment analysis revealed specific dysregulated biological processes as well. Furthermore, proteins with altered expression unique to a specific lung cancer type were identified and could be the targets of future studies.

## 1. Introduction

Lung cancer (LC) is one of the most frequently diagnosed cancers, responsible for 11% of new tumor cases in 2020, and it is the leading cause of cancer-related deaths. The 5-year relative survival rate for all stages combined is 21%. This low survival rate is consistent with more than half of the patients being diagnosed with metastatic disease [1,2]. The investigation of molecular changes in LC is crucial for exploring the mechanisms of tumor development, as well as the identification of novel therapeutic targets, markers for early detection, accurate disease prognosis, and ideal therapy selection.

Lung cancer is a heterogeneous disease with several known mutations and dysregulated signaling pathways. The World Health Organization (WHO) classifies it histologically into groups and several subgroups [3]. Small cell lung cancer (SCLC) and non-small cell lung cancer (NSCLC) are among the most common types of LC, accounting for 15% and 85%, respectively. The main subtypes of NSCLC are adenocarcinoma (AC), squamous cell carcinoma (SqCC), and large cell carcinoma (LCC), accounting for approximately 40%, 25–30%, and 5–10% of all cases [4]. The classification of LC has important therapeutic implications [5], which makes it a crucial part of the diagnosis. The original classification based on morphology has been improved by the utilization of protein markers such as thyroid transcription factor, p40, cytokeratin 5/6, and oncogenes as epidermal growth factor receptor (EGFR), or anaplastic lymphoma kinase (ALK) [6].

Current studies are mainly focused on the discovery of early diagnostic, prognostic, and predictive biomarkers of LC via genomics [7,8] and proteomics [9,10]. Compared to DNA markers, protein markers have the advantage that the biological processes involved in tumorigenesis and progression are exerted directly through them. Proteomics is a highly efficient tool for the identification of biomarkers, therapeutic targets, and exploring mechanisms of tumor development and progression. Ultra-high-performance liquid-chromatography coupled to tandem mass spectrometry (UHPLC-MS/MS) is the most suitable apparatus for in-depth proteomic analysis.

Numerous reviews have been published describing potential novel biomarkers for LC identified by proteomics studies, such as GRIP and coiled-coil domain-containing protein 2 (GCC2), Cystatin A, macrophage migration inhibitory factor (MIF), Thymosin β4, and Fascin [9,10]. Most potential blood [11,12,13] and saliva [14] markers identified for early diagnosis have not been implemented in clinical practice yet. The analysis of fresh-frozen or formalin-fixed paraffin-embedded (FFPE) tissue specimens/sections provides the basis of cancer research; several studies have been published previously about the proteomic characterization of NSCLC tissue [15,16,17,18]. On-surface digestion of these tissue sections further increases the efficiency of the proteomic analysis when limited sample material is available [19,20].

Our goal was to identify dysregulated biological processes by analyzing and comparing the four main types of LC by proteomics using on-surface tryptic digestion of FFPE tissue, enabling a more precise diagnosis and classification of lung cancer.

## 2. Materials and Methods

### 2.1. Materials

All chemicals used were HPLC-MS grade. Acetonitrile (ACN), water, acetone, formic acid (FA), ammonium-bicarbonate, citric acid, trisodium citrate, and heptafluorobutyric acid (HFBA) were purchased from Merck (Darmstadt, Germany). Trifluoroacetic acid (TFA), dithiothreitol, and iodoacetamide were obtained from Thermo Scientific (Unicam, Budapest, Hungary). Methanol was purchased from VWR International (Debrecen, Hungary) and RapiGest surfactant was obtained from Waters (Budapest, Hungary).

### 2.2. Tissue Sample Selection

Formalin-fixed paraffin-embedded (FFPE) AC, SqCC, LCC, SCLC (*n* = 10, 9, 10, 9, respectively) and tumor-adjacent normal tissue sections (*n* = 8, 8, 8, 9, respectively) were analyzed. The work was approved by the Medical Research Council (TUKEB permit number: IV/2567-4/2020/EKU).

Inclusion criteria for patients were the following: fresh LC cases with resection specimens, while also keeping in mind that histological groups should have similar sizes (our aim was 10 patients in each group). FFPE tissue sections with a thickness of 10 µm were obtained from the departmental archive of the Department of Pathology, University of Pécs, Hungary. Summarized information on the samples are provided in Table 1; for detailed information see Table S1.

### 2.3. On-Surface Digestion

Tissue sections were baked at 60 °C for 2 h to prevent tissue detachment. Next, deparaffinization was carried out by sequentially incubating the slides in xylene for 2 × 3 min, in ethanol for 2 × 5 min, in 90:10 *v*/*v*% ethanol:water for 3 min, in 70:30 *v*/*v*% ethanol:water for 3 min, in 10 mM NH_4_HCO_3_ (water) for 5 min, and, finally, in water for 1 min. After dewaxing, heat-induced antigen retrieval was performed (95 mM trisodium citrate + 21 mM citric acid in water, pH = 6) for 30 min at 80–85 °C to disrupt cross-linking induced by formalin fixation.

Following the preparation steps, digestion was carried out on specific tissue regions based on characterization by a pathologist. The proteins were reduced using 0.1% RapiGest and 5 mM dithiothreitol in 3 µL of 20% glycerol for 20 min at 55 °C, then alkylated using 10 mM iodoacetamide in 3 µL of 25 mM ammonium bicarbonate (ABC) buffer and 20% glycerol for 20 min at room temperature in the dark. The digestion was performed cyclically, each one lasting for 40 min at 37 °C in a humidified box with 5 cycles in total. In the first two cycles, LysC-Trypsin mixture was added in ca. 1:25 ratio, in 3 µL 50 mM ABC and 20% glycerol. Subsequently, in the last three cycles, Trypsin was added in a 1:5 ratio, in 3 µL 50 mM ABC, and 20% glycerol. After the digestion steps, the extraction of the protein digest was carried out by pipetting 3 µL 10% acetic acid extraction solvent five times on the digested spots. Peptide extracts were then dried down and stored at −20 °C until further usage.

### 2.4. Reversed-Phase Purification

C_18_ spin columns were used for desalting and clean-up. After the column was conditioned, washed, and equilibrated, the sample was loaded onto the column in 0.1% HFBA in water. The elution was performed with 30:70 *v*/*v*% water:ACN. After the elution, the samples were dried down and stored at −20 °C until further usage.

### 2.5. nanoUHPLC-MS(MS) Analysis

Samples were analyzed using a Maxis II QTOF instrument equipped with a CaptiveSpray nanoBooster ion source coupled to a Dionex UltiMate 3000 RSLCnano system (Bruker Daltonics GmbH, Bremen, Germany). Peptides were separated on an Acquity M-Class BEH130 C_18_ analytical column (1.7 μm, 75 μm × 250 mm, Waters, Budapest, Hungary) using gradient elution (isocratic hold at 4% for 11 min, then elevating B solvent content to 25% in 75 min, and to 40% in 15 min) following trapping on an Acclaim PepMap100 C_18_ (5 μm, 100 μm × 20 mm, Thermo Fisher Scientific, Waltham, MA, USA) trap column. Solvent A consisted of 0.1% FA in water, Solvent B was 0.1% FA in ACN, and the sample loading buffer was 0.1% TFA + 0.01% HFBA in water.

For MS analysis, DDA measurements were performed. The cycle time was set at 2.5 s, with a dynamic MS/MS exclusion of the same precursor ion for 2 min, or if its intensity was at least 3 times larger than before. Preferred charge states were set between +2 and +5. MS spectra were acquired at 3 Hz in the 150–2200 *m*/*z* range, while MS/MS spectra were at 4 or 16 Hz, depending on the intensity of the precursor. Internal calibration was performed by infusing sodium formate and raw data was recalibrated using the Compass DataAnalysis software 4.3 (Bruker Daltonics, Bremen, Germany).

### 2.6. Software

Software used: Byonic 3.8 [21] (https://proteinmetrics.com, accessed on 22 April 2022 MaxQuant 1.6.17 [22] (https://maxquant.org, accessed on 22 April 2022), R 3.6.1 [23] (https://www.r-project.org/, accessed on 22 April 2022),), RStudio 1.2.5001 [24] (https://rstudio.com/, accessed on 22 April 2022), GSEA 4.2.3 [25] (https://www.gsea-msigdb.org/gsea/index.jsp, accessed on 22 April 2022), Cytoscape 3.9.1 [26] (https://cytoscape.org/, accessed on 22 April 2022). The exact parameters used for all the software are summarized in Table S2.

### 2.7. Data Analysis

Protein quantitation was performed in MaxQuant on a focused Homo Sapiens database, made from merging Byonic search results from all MS/MS analyses. Subsequent data analysis steps were performed in R using RStudio. After quantitation, the data were filtered based on the number of missing values in each of the eight sample groups: only proteins found in at least 2/3 of all samples in at least one sample group were kept for further analysis. Missing values were then imputed in a group-wise manner according to the following: if the protein in question was found in less than 2/3 of all samples in the group, it was imputed with the sample 5-percentile, while if it was found in at least 2/3 of all samples in the group, it was imputed according to the kNN algorithm (VIM package [27], k = 15, similarity based on Euclidean distances, default settings used). Following imputation, statistical analysis was carried out. For the different comparisons (multiple and two-group comparisons) within a group, normality and equality of variances were tested (Shapiro–Wilk tests and Levene tests, respectively). For multiple group comparisons, Analysis of variance (ANOVA), Welch-ANOVA, and Kruskal–Wallis tests, for two-group comparisons, Student’s *t*-tests, Welch *t*-tests, and Wilcoxon rank sum tests were performed based on the outcome of the normality and variance equality tests. False discovery rates were controlled for all two-group and all multiple-group comparisons separately using the Benjamini-Hochberg method at 5%. Plots were made using the ggplot2 [28], ggpattern, gplots, and nVennR [29] packages. The principal component analysis (PCA) was performed using the prcomp function (using variable scaling and default settings), hierarchical clustering was performed using the heatmap.2 function (using Ward’s clustering method “ward.D2” from the hclust function). The identification of enriched gene sets was performed with the GSEA software using the GSEAPreranked function based on effect sizes (Cohen’s *d*) calculated for the four types of LC separately (adjacent vs. tumorous tissue). For gene sets, the Hallmark, KEGG, and GO databases available in GSEA were used.

### 2.8. Data Availability

Experimental data were submitted to the MassIVE data repository with the ID: MSV000089291.

## 3. Results

The 71 samples analyzed were derived from FFPE tissue sections taken from individuals suffering from either of the four different types of lung cancer (AC, SqCC, LCC, SCLC). From the tissue sections, samples were analyzed from both the cancerous and the cancer-adjacent regions (based on histopathological characterization). However, this was not possible in cases where there was only tumor tissue present, hence the group sizes are not equal; 8–10 samples belong to each sample group (patient and sample information is summarized in Table 1). Of the ten samples with large cell morphology, three were large cell neuroendocrine carcinoma (LCNEC) according to the diagnostic criteria of the latest WHO classification. LCNEC is part of the neuroendocrine carcinoma group (beside the more common small cell neuroendocrine carcinoma), but because of the small size of the LCNEC group, and the similarities in microscopical morphology, these three LCNECs are grouped with the LCC in the current study.

During the initial protein identification step—used for the construction of a focused protein database—9316 different proteins were identified altogether by Byonic software, on average ca. 1600 from the individual samples, as peptides from 3 µL digestion areas on the tissue surface were extracted and analyzed. Using the focused database, 2917 proteins were quantified by MaxQuant using label-free quantitation, out of which 1345 were considered for statistical analysis after initial filtering (exact methods used for data analysis and statistics are summarized in Table S2).

**Figure 1:**
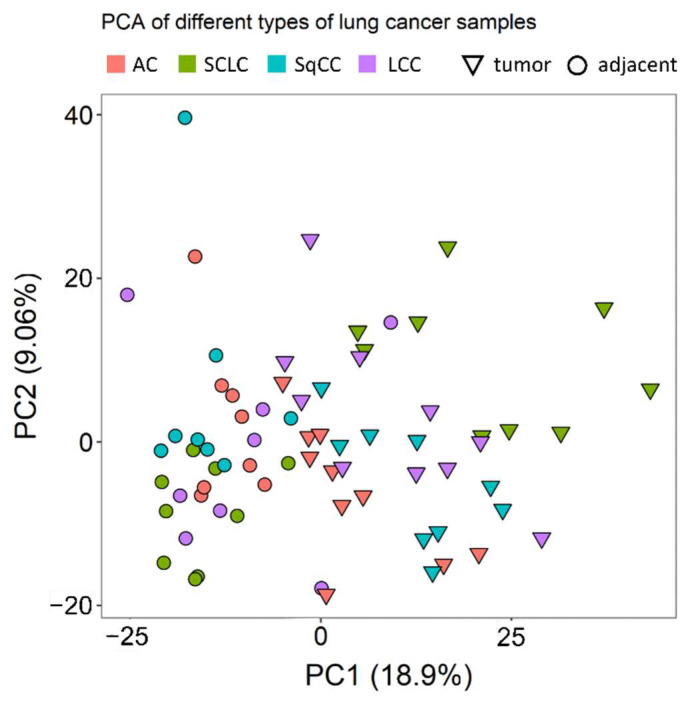
PCA of the different samples analyzed. Triangles and circles indicate tumor and tumor-adjacent samples, respectively. Different colors mark different LC types (red for AC, green for SCLC, turquoise for SqCC, and purple for LCC).

Principal component analysis (PCA) showed that there are considerable differences between the tumorous and the tumor-adjacent regions, especially when considering a single LC type at a time (Figure 1).

Following the initial assessment, the data were further investigated to identify proteins that are differentially expressed between (i) all adjacent and all tumor tissue, (ii) adjacent and tumor tissue in each type, and (iii) the different types of tumor tissue.

**Figure 2:**
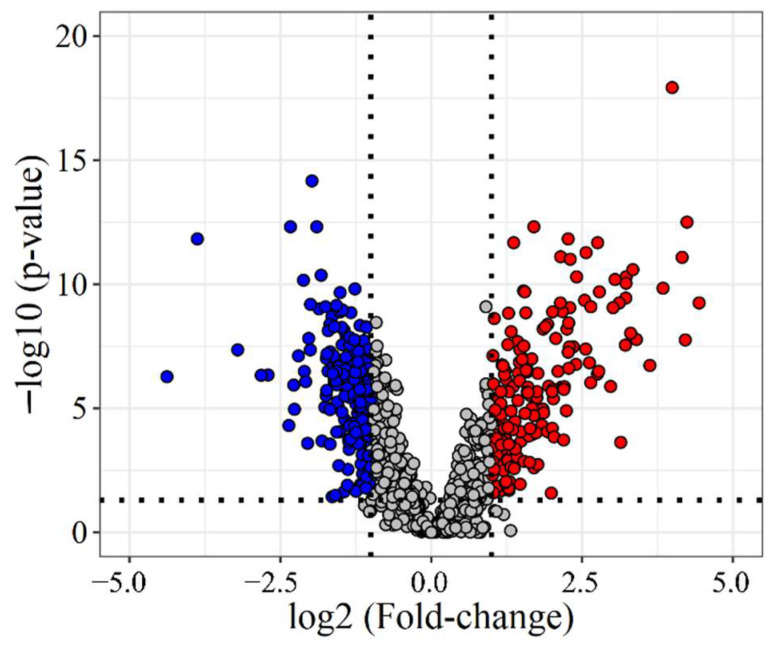
Volcano plot of all quantified proteins. Fold-change and *p*-values were calculated between all tumor and all adjacent tissue samples. Blue—significantly under-expressed in LC (FC < 0.5), red—significantly overexpressed in LC (FC > 2).

To identify differences between adjacent and tumor tissue (33 and 38 samples, respectively), two-group comparison tests were used (for details see Methods). Based on these, 845 proteins were found to be differentially expressed, 356 with a fold-change (FC) of over 2 (Figure 2), 183 under-expressed, and 173 overexpressed in LC. For example, several components of the basement membrane (e.g., Collagens, Nidogen-1, Laminin subunit 3) were downregulated in tumor tissue, and proteins involved in Calcium-ion binding (e.g., Annexin-A3, S100A4) showed lower expression levels in tumor tissue than in tumor-adjacent tissue. Additionally, many proteins related to Ribonucleoprotein biogenesis and organization (Small- and Large ribosomal subunit proteins) were overexpressed in tumor tissue.

Two-group comparisons were performed separately for all 4 LC types. This revealed that there are 78 proteins differentially expressed in all four LC types compared to adjacent tissue (For an excerpt, see Table 2; the complete list is included in Table S3).

**Figure 3:**
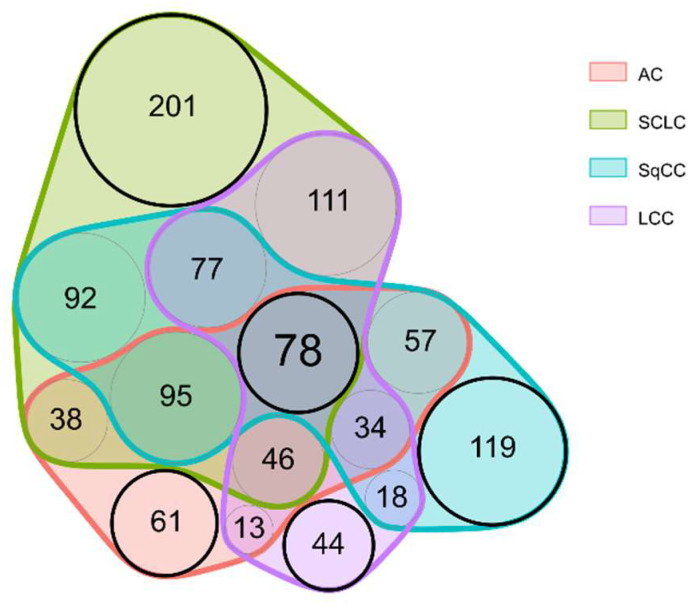
Venn diagram displaying the proteins differentially expressed in the different LC types compared to adjacent tissue.

The majority of alterations occurred in a group-specific manner: 61 proteins were differentially expressed only in AC, while 201, 119, and 44 proteins only in SCLC, SqCC, and LCC, respectively (for a Venn diagram displaying these differences, see Figure 3; for a comprehensive protein list, see Table S4). For example, proteins connected to the hemostasis (e.g., Fibrinogens), and proteins involved in the RHO protein signal transduction (e.g., Apoliproteins, CO1A2) showed lower expression levels only in SqCC tissue compared to tumor-adjacent tissue. Furthermore, several proteins related to splicing (splicing factors, and heterogeneous nuclear ribonucleoproteins) were significantly overexpressed only in SCLC tissue, while proteins related to microtubule organization (e.g., TBB4B, TBB5, MAP4, and MAP1S) were also found to be highly overexpressed only in SCLC tissue.

**Figure 4:**
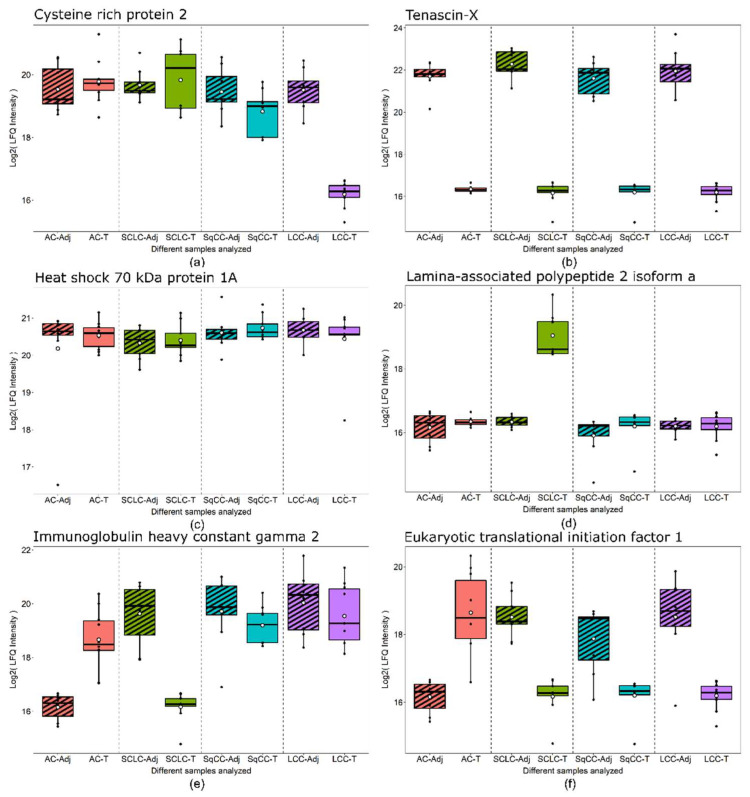
Box plots of protein examples for distinct expression patterns observed. (**a**) Cysteine-rich protein 2 showed under-expression in only LCC tumor tissue. (**b**) Tenascin-X was under-expressed in all tumor tissue types compared to adjacent tissue. (**c**) Heat shock 70 kDa protein 1A did not show differential expression between any tissue-pairs. (**d**) Lamina-associated polypeptide 2, isoform alpha was overexpressed in only SCLC tumor tissue. (**e**) Immunoglobulin heavy constant gamma 2 was overexpressed in AC tumor, while under-expressed in SCLC tumor compared to adjacent tissue. (**f**) Eukaryotic translational initiation factor 1 was overexpressed in AC tumor while under-expressed in all other tumor types compared to adjacent tissue. Boxes that represent adjacent tissue are striped, while tumor tissue are filled without pattern.

The specific groups highlighted in Figure 3 are particularly noteworthy. First, the „central” 78 proteins (Table S3) might be useful in detecting LC, regardless of type. Second, the other „LC type-specific” proteins might be used for the detection of different types of LC following further validation. Six examples are presented in Figure 4 showing examples of proteins showing distinct behavior patterns. Cysteine-rich protein 2 was under-expressed in only LCC tumor compared to the tumor-adjacent normal tissue regions and is subsequently part of the 44 proteins differentially expressed only in LCC tissue (Figure 3). Tenascin-X, on the other hand, was under-expressed in all types of LC analyzed; thus, part of the 78 proteins differentially expressed in all tumor types (Figure 3). Heat shock 70 kDa protein 1A did not show differential expression but was highly abundant in all tissue types. Lamina-associated polypeptide 2, isoform alpha was overexpressed only in SCLC tumor tissue and is part of 201 proteins differentially expressed only in SCLC (Figure 3). Finally, Immunoglobulin heavy constant gamma 2 and Eukaryotic translational initiation factor were found to be differentially expressed in more than one LC types, however, not in a uniform direction.

We identified the proteins differentially expressed between the cancerous tissue regions of the four types of LC using multiple sample and two-sample comparison tests (for details see the Methods section), resulting in 571 proteins with altered expression (Table S5). None of the proteins showed altered expression in all pairwise comparisons: 23 of them showed differences in 5 out of 6 comparisons (Table 3). There were several proteins with changes in expression in only one LC type, separating it from all the others (61 for AC, 99 for SCLC, 35 for SqCC, and 47 for LCC). For example, Eukaryotic translation initiation factor 1 and Matrix-metalloproteinase proteins (MMP2 and MMP19) were found to be upregulated in AC tissue compared to other LC types. IgG heavy constant gamma 2, and RAB10—a small GTPase related to Golgi vesicle transport—were significantly dowregulated in SCLC tissue compared to other LC types. Fascin—an actin filament bundling protein—was found to be significantly overexpressed in SqCC tissue.

**Figure 5:**
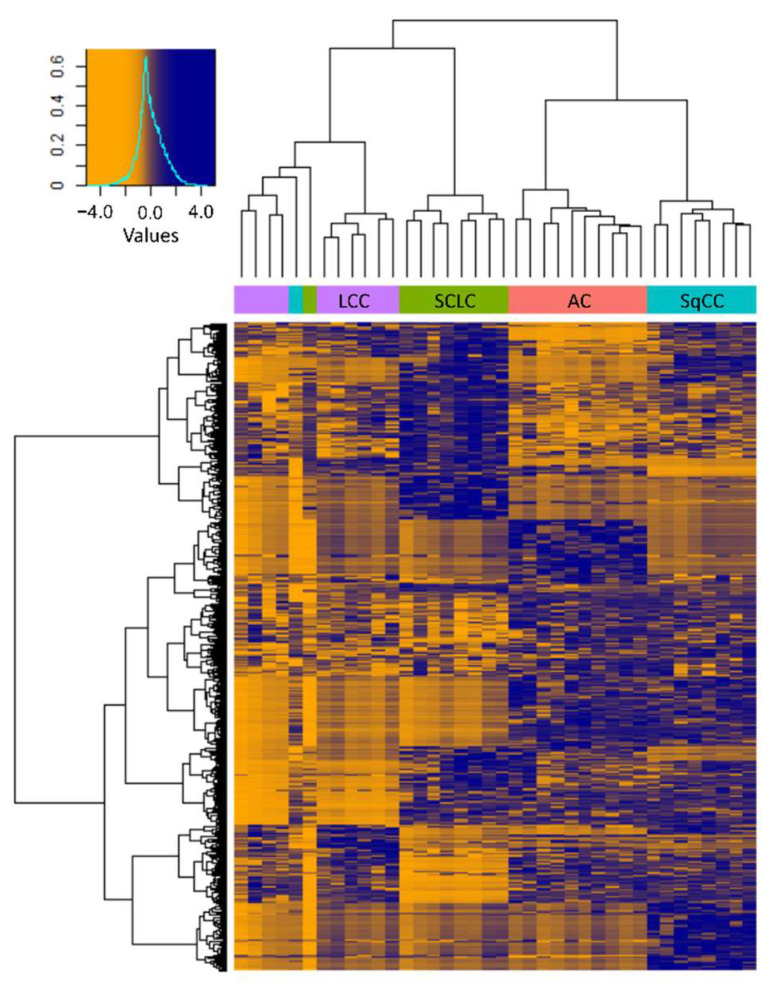
Heatmap with hierarchical clustering of the 571 proteins showing differential expression between the tumorous regions of the 4 LC types investigated (Protein LFQ Intensity values are Z-scored). The histogram in the top left corner shows the correspondence between color hues and values.

Hierarchical clustering based on the 571 differentially expressed proteins revealed that the molecular profiles of the different types of LC were markedly different, and the clustering of samples—except for 2—was in agreement with the pathological classification (Figure 5). Despite the histological classification, the three LCNEC samples clustered closely together with the 7 LCC samples, confirming that the phenotype is determined by a more complex molecular profile.

**Figure 6:**
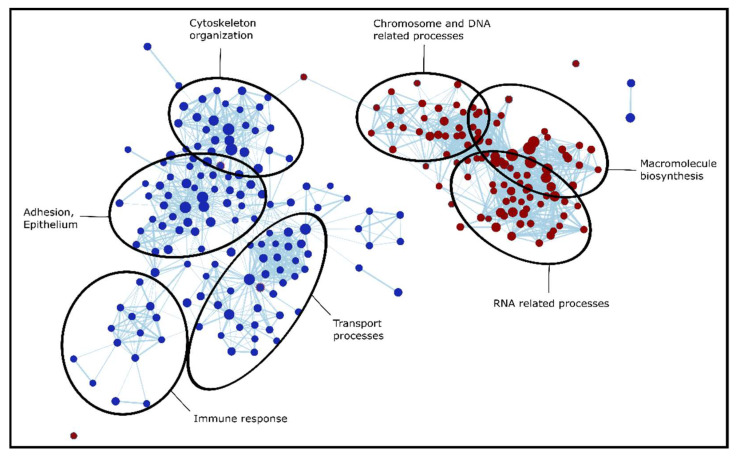
Interaction network of gene sets based on the GSEA results for Gene Ontology Biological Process terms enriched in SCLC. Nodes representing suppressed gene sets are colored blue, while red for elevated gene sets. Furthermore, node sizes indicate the number of genes (proteins) detected.

To identify the dysregulated biological processes in the tumorous regions compared to adjacent tissue, we applied pre-ranked Gene Set Enrichment Analysis (GSEA). This was performed separately for all four LC types (based on effect sizes included in Table S6), then the results were compared to reveal gene sets enriched in LC in general, and also in a type-specific manner (Table S7). When discussing and comparing GSEA results, the Normalized Enrichment Score (NES) is reported occasionally, which is a normalized score that represents the degree to which a gene set is overrepresented on the top or the bottom of the gene list. The enriched gene sets could be separated into several major groups of biological processes: extracellular matrix (ECM) organization, assembly, regulation and adhesion; signaling cascades (e.g., Ca^2+^ dependent signaling, RHO signaling); processes involved in protein synthesis (transcription, translation, DNA, and RNA related processes); humoral and cell-mediated immune system processes; and transport processes (e.g., vesicular transport, endocytosis). The processes dysregulated in LC were similar in all types; however, there were several type-specific differences, especially between SCLC and NSCLC. This is not surprising based on Figure 3, which shows that SCLC has the largest number of unique dysregulated proteins out of all 4 types investigated. Biological processes altered in SCLC tissue are visualized in Figure 6: cytoskeleton organization, adhesion, immune response, and transport processes are highly suppressed in SCLC, while chromosome, DNA, RNA related processes, and macromolecule biosynthesis are enriched in SCLC tissue compared to adjacent tissue.

## 4. Discussion

The four most common types of lung cancer (AC, SCLC, SqCC, and LCC) have been investigated through MS-based proteomic analysis of FFPE tissue sections following on-surface digestion. To the best of our knowledge, this is the first pilot study comparing these four types of LC in the same cohort. On-surface digestion has the advantage that small tissue areas corresponding to cancerous and cancer adjacent regions can be investigated from the same FFPE material. However, there is a compromise, as analyzing small tissue areas results in fewer proteins identified compared to bulk tissue analysis. Our previous results indicate that this approach can be successfully used for FFPE tissues and biopsies [20]. We compared our results to a study specifically focused on lung AC, and we have found that all of the 12 proteins listed in Table 2 were all detected in both studies and the direction of changes were in agreement [30]. Furthermore, two of them (Annexin A3 and Tenascin-X) were previously reported with altered blood levels between LC patients and healthy controls [31].

The grouping of samples in the current pilot study was based on histological appearance. Three tissues grouped among LCC samples were LCNEC based on classification, as they expressed at least one out of several neuroendocrine markers. The phenotype of LCNEC cells, however, is influenced by their entire molecular profile, not just the expression of specific markers, therefore we grouped them with LCC samples. Furthermore, based on hierarchical clustering (Figure 5), these three LCNEC samples clustered with the LCC samples, confirming the validity of our grouping based on large cell morphology.

Identified proteins with altered expression levels—comparing any of the types or adjacent samples—will be discussed in detail, such as the results of the gene set enrichment analysis which was performed to identify altered biological processes. The biological pathways discussed and the direction of their dysregulation are summarized in Table 4. Considering that we have analyzed the cancerous and tumor-adjacent normal regions of these tissues, some proteins and biological processes which are otherwise dysregulated in cancerous tissue compared to healthy controls may not have been revealed. The main biological processes identified as disrupted in any of the types of LC analyzed in our pilot study include extracellular matrix remodeling, altered adhesion, signaling cascades, immune response, coagulation, protein biosynthetic processes, metabolic processes, and vesicular transport.

### 4.1. Extracellular Matrix Remodeling

Extracellular matrix (ECM) remodeling processes are prevalent in cancer, creating a microenvironment that promotes tumorigenesis and metastasis [32]. We have identified through GSEA analysis that the gene ontology cellular compartment (GOCC) gene set *Collagen containing ECM* was significantly and heavily suppressed in all four LC types investigated (with an average NES value of over 2). This is, in the most part, due to the altered expression of major ECM constituent protein families: collagens, laminins, nidogens, proteoglycans, and matrix metalloproteinases.

We have quantified 17 collagen proteins altogether. None of them showed altered expression between the four cancer tissue types, but most of them were downregulated in cancerous tissue in general, compared to adjacent tissue. Several types of collagens play a complex role in tumor proliferation, invasion, angiogenesis, and metastasis [33,34]. Elevated levels of CO1A1 have been reported to be associated with chemoresistance and poor progression-free survival in metastatic lung cancer [35]. Nidogen-1 (NID1) was downregulated in all tumor types, while NID2 was downregulated only in SCLC, compared to adjacent tissue. It has been previously reported that in serum samples, the degradation of NID1 is associated with NSCLC. It was also reported that NID1 enhances cell proliferation, migration, invasion, and promotes lung metastasis of breast cancer and melanoma.

We have quantified seven subunits of laminin. Subunit gamma-1 (LAMC1) was downregulated in all four cancer types, while all other subunits showed lower expression levels in SCLC; LAMC2, LAMA5, LAMB2 in LCC; and LAMA5, LAMB2, LAMA3 in AC as well. Additionally, LAMB1 was under-expressed in SCLC and LCC but overexpressed in SqCC, and it also showed significant differences between SqCC and all other tumor tissues. Moon and coworkers suggest that LAMC2 promotes metastasis in AC [36], while another study demonstrated the significance of circulating LAMC2 as a prognostic marker in SCLC, especially for early stage cancer [37].

Out of the nine proteoglycans quantified in our samples, Versican was overexpressed in AC, while Perlecan, Decorin, Prolargin, and Mimecan were all under-expressed in SCLC and LCC. Agrin and Lumican were downregulated in SCLC: Biglycan showed lower expression levels in AC, SCLC, and SqCC. The versatility of changes in proteoglycan expression levels in the different types of LC is in line with previous studies, which report that their role in cancer is highly context-dependent [38,39].

We have identified five different matrix metalloproteinases (MMPs). MMP-2 was upregulated in AC compared to both other cancer types and adjacent tissue, making it a promising target for future studies. MMP-12 was significantly overexpressed in SCLC and LCC, MMP-19 was overexpressed in AC but under-expressed in SCLC. Based on previous studies, the overexpression of MMP2 is associated with tumor differentiation, invasion, angiogenesis, and metastasis [40].

### 4.2. Altered Adhesion, Cytoskeleton Remodeling

GSEA analysis revealed that many gene sets involved in adhesion and cytoskeleton remodeling were dysregulated [41,42,43]. The gene ontology biological process (GOBP) gene sets *Regulation of cell adhesion* (and related terms such as cell-matrix, and cell-substrate adhesion) was suppressed in SCLC and SqCC; the gene ontology molecular function (GOMF) gene sets *Actin binding* in SCLC, SqCC and LCC; and *Collagen, and Integrin binding* in AC, SCLC, and SqCC. The GOCC gene sets involved in cytoskeleton formation were also suppressed, e.g., *Actin cytoskeleton* in SCLC, SqCC, and LCC, and *Microtubule cytoskeleton* in SCLC. Finally, the KEGG gene set *Focal adhesion*—a well-known process involved in cancer metastasis [44]—was found to be heavily suppressed in all types of LC. These changes are due to the altered expression (mostly under-expression) of proteins involved in cytoskeleton formation and cell adhesion, e.g., actins, tubulins, microtubule-associated proteins, spectrins, and galectins.

Actin, cytoplasmic 2 (ACTG) and actin, alpha cardiac muscle 1 (ACTC) were found to be significantly under-expressed in SCLC and LCC. Furthermore, most of the identified actin-related proteins, such as myosins and filamins, were heavily downregulated in SCLC tissue. On the other hand, Fascin (FSCN1)—an actin filament bundling protein—was significantly overexpressed in SqCC tissue compared to the other cancer types. The overexpression of FSCN1 in NSCLC has been previously reported to be associated with tumor growth, migration, invasion, and metastasis [45,46].

All of the five identified tubulins showed altered expression in the different types of LC compared to adjacent tissue. We have also identified changes in the expression of microtubule-associated proteins (MAPs). Microtubule-associated protein 1B (MAP1B) was significantly upregulated in SCLC tissue compared to the different types of NSCLC and adjacent tissue. In addition to MAP1B, we identified other dysregulated microtubule-associated proteins, MAP1S and MAP4. MAPs have been previously reported to be commonly overexpressed in cancer [41,42].

Spectrins were also found to be altered in LC, although there was a big difference between SCLC and NSCLC in this regard. All of them showed under-expression in SCLC while only 1 in each of the other NSCLC types. Furthermore, spectrin beta chain (SPTB) also showed differential expression between SCLC and all other cancer tissue. We have also identified Ankyrin-1 (ANK1)—a protein that regulates cell shape and membrane integrity together with spectrins—to be downregulated in all LC types. It has been previously reported that ANK1 and spectrins—such as SPTB and SPTA1—were under-expressed in AC compared to paired non-malignant tissue [47].

We have identified two galectins (LEGs) in our samples. We have found that LEG1 was significantly under-expressed in SCLC compared to SCLC adjacent tissue. Expression levels of LEG3 in SCLC and LCC tumor were significantly lower than in AC, SqCC, and adjacent tissue. LEG1 promotes tumor cells invasion and migration; it is also a potential prognostic marker in early stage NSCLC [48] and is feasible for the promotion of chemoresistance in AC [49]. Furthermore, LEG1 and LEG3 are associated with LC and correlated with tumor invasion, migration, metastasis, and progression [49].

### 4.3. Signaling Cascades

Many signaling pathways have been identified as dysregulated in LC tissue. The *Mammalian target of Rapamycin 1 (MTORC1) signaling pathway*—represented by the hallmark gene set—was found to be elevated in all four types of LC. The MTORC1 pathway plays an important role in regulating fatty acid (FA) metabolism, and, subsequently, the production of ATP. Furthermore, we found that ATP-citrate synthase (ACLY)—a key enzyme involved in Acetyl-CoA synthesis regulated by MTORC1—is significantly overexpressed in AC. The overexpression of ACLY has been observed in multiple tumor types before [50]. It has also been shown that targeting ACLY significantly reduces the growth of lung and prostate tumor xenografts [51].

The GOBP gene set *RHO protein signal transduction* was found to be significantly suppressed in SqCC. From the gene set, three corresponding proteins were found to be highly under-expressed in SqCC compared to adjacent tissue: Apolipoprotein A-I (APOA1), Apolipoprotein E (APOE), and Collagen alpha-2(I) chain (CO1A2). APOA1 has been previously reported to be under-expressed in NSCLC serum and associated with metastasis and poor prognosis [52]. APOE is an important marker of cancer, its overexpression in NSCLC has been reported before. Downregulation of CO1A2 has also been reported previously in NSCLC [53].

The GOMF gene set *Calcium ion binding* was found to be significantly and highly suppressed in SCLC. This is, in a large part, due to the changes we detected in the expression levels of Annexins—a large family of Ca^2+^-binding proteins involved in signaling processes. We have quantified seven different Annexins with highly diverse expression profiles between the different types of LC. Annexin A3 (ANXA3) was found to be under-expressed compared to adjacent tissue in all types of LC. Aberrant expression of ANXA3 has been previously reported to promote tumor cell proliferation, invasion, metastasis, angiogenesis, and therapy resistance [54]. ANXA4 was under-expressed in all types except for AC. Previous studies suggest that ANXA4 enhances tumor invasion and promotes anti-tumor drug resistance [55]. Our data also show that ANXA2 was under-expressed in SCLC and LCC. Out of the seven Annexins quantified, five showed differential expression in SCC, three in LCC, two in SqCC, and only one in AC. We found Protein S100-A4—another Ca^2+^-binding protein—to be under-expressed in all types of tumor tissue. It was previously reported to be downregulated in tumor cells compared to stromal non-tumor cells, confirming our results [56].

### 4.4. Immune Response

Immune system processes are often deregulated in cancer. It has also been demonstrated that inflammatory immune cells are essential players in cancer-related inflammation [57]. We have identified multiple processes involved in both humoral and cell-mediated immune responses, as altered in the different LC types analyzed. The KEGG gene set *Complement and coagulation cascade* was found to be suppressed in all four types of LC tumor tissue. On the other hand, humoral immune response and biological processes under cell-mediated immune response—*Leukocyte mediated immunity, Lymphocyte mediated immunity, B cell mediated immunity, and Phagocytosis*—were suppressed only in SCLC and SqCC. These LC type-specific differences were highlighted by the differences in the expression of the 12 Immunoglobulin G (lgG) proteins identified. Nine of them were significantly differentially expressed in SCLC, while only three in AC, four in SqCC, and two in LCC. Furthermore, there were substantial differences in the direction of these changes. Previous studies have also reported differences in IgG expression and subclass distribution in LC patients [58,59].

### 4.5. Hemostasis

Pathophysiological changes in the body can affect the hemostatic system. Specifically, LC has been reported as one of the cancer types with the most risk of developing venous thromboembolism [60]. We have found that the GOBP gene sets, *Hemostasis* and *Regulation of coagulation,* were suppressed in AC, SCLC, and SqCC, however, in SqCC, with an especially high NES (2.2 and 2.8, respectively). This is primarily due to the differences in fibrinogen expression levels. We identified three fibrinogen proteins: Fibrinogen alpha chain (FIBA), Fibrinogen beta chain (FIBB), and Fibrinogen gamma chain (FIBG). In SqCC, all of them were under-expressed, while none of them in the other types of LC. The importance of fibrinogens in cancer is highlighted by the fact that plasma fibrinogen levels have been previously suggested as prognostic markers in NSCLC, combined with the neutrophil-to-lymphocyte ratio [61,62].

### 4.6. Regulation of Protein Biosynthetic Process

Increased protein synthesis and subsequent rapid cell growth is one of the hallmarks of cancer, which can be activated through the dysregulation of various biological processes [63]. We have identified several of these connected to four major umbrella terms: translation, mRNA, ribonucleoprotein, and chromosome-related processes.

The GOBP gene set *Cytoplasmic translation* was found to be activated in all four types; on the other hand, *Translational initiation* was elevated only in AC, SCLC, and SqCC. This is explained by the differences in the expression levels of the 16 initiation factors (IFs) identified. The number of IFs differentially expressed were the lowest in LCC compared to other types of LC, with significantly lower effect sizes. Three initiation factors showed differential expression in all four types: Eukaryotic initiation factor 4A-I (IF4A1) and Eukaryotic translation initiation factor 6 (IF6) showed overexpression in all four; however, Eukaryotic translation initiation factor 1 (EIF1) was under-expressed in SCC, SqCC and LCC, but overexpressed in AC. EIF1 also shows a significant difference in expression between AC and all other types The dysregulation, specifically the overexpression of the initiation factors, has been reported previously to occur frequently in cancer [64].

We identified multiple GOBP gene sets involved in mRNA processing, elevated in all four types: *mRNA* and *ncRNA processes, mRNA* and *ncRNA metabolic processes*, and *Gene silencing by RNA*. However, gene sets related to splicing: *Alternative mRNA splicing*, and *Regulation of mRNA splicing* via *spliceosome*, were elevated only in SCLC. This can be explained by looking at the expression levels of Splicing factors (SFs), and Heterogeneous nuclear ribonucleoproteins (hnRNPs). Out of the 16 SFs identified, we detected the significant overexpression of 13 in SCLC, while in the case of the NSCLC, subtypes this number were much lower. Regarding the 15 hnRNPs, 13 showed significant overexpression in SCLC, while—similarly to SFs—this number was much lower in the NSCLC subtypes. mRNA-related processes are potential therapeutic targets for cancer, as it has been reported that alterations in mRNA can contribute to the initiation and progression of cancer [65]. We detected the activation of GOBP gene sets *Ribonucleoprotein biogenesis* and *Ribonucleoprotein subunit organization* in all four types of LC. We identified 18 Small ribosomal subunits (40S) and 27 Large ribosomal subunits (60S) proteins, with many of them overexpressed in all four types of LC. The altered expression of Ribosomal proteins has been linked to multiple LC-related processes, e.g., the downregulation of 60S ribosomal protein L3 (uL3) has been linked to drug resistance in LC cells, and 40S ribosomal protein S6 (eS6) was found to be overexpressed in NSCLC [66,67].

Other important processes connected to protein biosynthesis that we found to be activated in LC are *Regulation of chromosome organization* (in SCLC, SqCC, and LCC) and *Chromatin organization* (in SCLC) and *Positive regulation of DNA metabolic process* and *Positive regulation of DNA repair* (in SCLC, SqCC, and LCC). It has been previously reported that chromosome loop structures can be altered in cancer and contribute to oncogene dysregulation [68]. Furthermore, it has been suggested that DNA repair pathways could be used as therapeutic targets in both SCLC and NSCLC [69].

### 4.7. Metabolic Processes

Metabolic remodeling is necessary for all stages of tumor development [63]. Several dysregulated GOBP gene sets involved in metabolic processes have been identified by GSEA. *Glycerolipid metabolic process* was elevated in AC, SCLC, and SqCC; *Pyruvate metabolic process* in AC, SqCC, and LCC; and *Cellular carbohydrate metabolic process* in AC and SqCC. This suggests that further investigation into the differences in metabolism between the different types of LC is reasonable, which is further highlighted by previous studies reporting that abnormal glycolysis and lipid metabolism have a significant role in the development of LC [70].

### 4.8. Vesicular Transport

We found that vesicle related GOCC and GOBP gene sets (e.g., *Endocytic vesicle, Regulation of vesicle mediated transport, Endocytosis*) were suppressed in SCLC and SqCC. It has been previously reported that LC-derived extracellular vesicles mediate epithelial-mesenchymal transition by the transfer of Vimentin—which we also found to be differentially expressed in SCLC and SqCC—and regulate angiogenesis, activate cancer-associated fibroblasts, and mediate metastasis [71,72]. We also found that *Golgi vesicle transport* was elevated in SqCC tissue but suppressed in SCLC. RAB10—a small GTPase of the RAS superfamily—is a key component of this process, which we found to be upregulated in SqCC and downregulated in SCLC. Rab GTPase proteins have been previously reported to have diverse roles in cancer progression as both oncoproteins and tumor suppressors [73].

## 5. Conclusions

In the frame of our pilot study, label-free quantitative proteomics followed by gene set enrichment analysis was performed on different lung cancer tissue types to identify proteins with altered expression and, subsequently, dysregulated biological pathways. Using our on-surface tryptic digestion approach, we identified several biological processes disrupted in all investigated cancer types, such as the degradation of the basement membrane and suppression of the complement and coagulation cascade, as well as the activation of the MTORC1 signaling pathway.

As expected, based on the proteomic profiles, differences between SCLC and NSCLC samples were larger than between the three distinct NSCLC subtypes. Dysregulated pathways differentiating SCLC from NSCLCs include suppressed regulation of cell adhesion, actin filament-based processes, and calcium ion binding. Overexpression of splicing factors and heterogeneous nuclear ribonucleoproteins suggest that biological processes connected to splicing are more affected in SCLC. Furthermore, the expression of several proteins showed changes only in one LC type, such as the overexpression of FSCN1 in SqCC or the downregulation of NID2 in SCLC. Our results correlated well with previous studies analyzing individual NSCLC types and tumor adjacent tissues, even though the tissue areas were smaller in the present pilot study. Although a relatively low number of samples were analyzed in the case of each LC subtype, and the validation of proteins has not been performed, these specific molecular signatures might be attractive targets for further in-depth investigations and can also bear potential diagnostic and prognostic value.

## Figures and Tables

**Table 1:** Summary of patient and sample information. In the case of large cell carcinoma, the numbers in parentheses represent the sum of large cell and large cell neuroendocrine carcinoma.

Sample Characteristics	No. of Patients	No. of Tumor Samples	No. of Tumor Adjacent Samples
Total No.	40	38	33
Age	66 (54–79)		
Gender			
Male	22	21	19
Female	18	17	14
Histology			
Adenocarcinoma	10	10	8
Large cell carcinoma	7 (10)	7 (10)	5 (8)
Large cell neuroendocrine carcinoma	3	3	3
Small cell lung carcinoma	10	9	9
Squamous cell carcinoma	10	9	8

**Table 2:** Excerpt of the proteins differentially expressed in all four types of LC compared to tumor-adjacent tissue. Proteins discussed in detail in further sections are listed.

Proteins	AC		SCLC		SqCC		LCC	
	*p*-Value	Log_2_FC	*p*-Value	Log_2_FC	*p*-Value	Log_2_FC	*p*-Value	Log_2_FC
IF6_HUMAN	2.26 × 10^−3^	1.9	2.85 × 10^−2^	1.1	1.47 × 10^−2^	2.5	2.34 × 10^−2^	1.5
HNRPF_HUMAN	1.06 × 10^−2^	1	8.84 × 10^−5^	1.6	8.69 × 10^−4^	1.3	2.48 × 10^−2^	1.2
IF4A1_HUMAN	2.45 × 10^−2^	1.4	3.83 × 10^−3^	1.4	3.93 × 10^−3^	1.5	2.98 × 10^−2^	1.2
CO6A2_HUMAN	3.08 × 10^−2^	−0.7	8.46 × 10^−4^	−2.5	1.87 × 10^−2^	−1.8	1.02 × 10^−2^	−1.7
CO6A1_HUMAN	7.84 × 10^−4^	−1.3	1.32 × 10^−4^	−2.1	9.33 × 10^−3^	−2.2	2.28 × 10^−2^	−2
ANXA3_HUMAN	1.16 × 10^−2^	−2.1	5.83 × 10^−4^	−4.1	2.34 × 10^−2^	−1.3	1.56 × 10^−2^	−2
ANK1_HUMAN	2.51 × 10^−3^	−2.3	3.91 × 10^−3^	−2.8	1.47 × 10^−2^	−2	1.31 × 10^−2^	−2.1
NID1_HUMAN	1.63 × 10^−2^	−2.4	2.89 × 10^−5^	−3.6	1.76 × 10^−2^	−2	1.89 × 10^−2^	−2.1
S10A4_HUMAN	2.54 × 10^−2^	−1.7	2.73 × 10^−3^	−2.3	5.83 × 10^−4^	−2.5	5.40 × 10^−3^	−2.1
EIF1_HUMAN	5.83 × 10^−4^	2.5	2.73 × 10^−3^	−2.3	2.34 × 10^−2^	−1.7	5.83 × 10^−4^	−2.3
LAMC1_HUMAN	2.02 × 10^−2^	−1.9	5.83 × 10^−4^	−3.9	6.44 × 10^−3^	−1.9	8.46 × 10^−4^	−2.5
TENX_HUMAN	2.78 × 10^−3^	−5.4	5.83 × 10^−4^	−6.1	8.46 × 10^−4^	−5.4	1.20 × 10^−8^	−5.8

**Table 3:** List of the 23 proteins differentially expressed between 5 out of 6 comparisons with *p*-values.

Proteins	AC vs. CLC	AC vs. SqCC	AC vs. LCC	SCLC vs. SqCC	SCLC vs. LCC	SqCC vs. LCC
S10A6_HUMAN	9.92 × 10^−5^	3.76 × 10^−2^	3.76 × 10^−2^	2.29 × 10^−3^	4.34 × 10^−2^	3.86 × 10^−1^
PCNA_HUMAN	1.39 × 10^−3^	2.41 × 10^−3^	1.09 × 10^−3^	2.53 × 10^−2^	2.64 × 10^−3^	8.38 × 10^−1^
MAP1B_HUMAN	2.47 × 10^−3^	2.10 × 10^−2^	2.91 × 10^−1^	2.85 × 10^−3^	4.34 × 10^−3^	2.98 × 10^−2^
FXR1_HUMAN	5.84 × 10^−4^	3.09 × 10^−2^	2.30 × 10^−3^	2.07 × 10^−1^	1.93 × 10^−8^	2.46 × 10^−5^
MCM6_HUMAN	1.30 × 10^−4^	2.47 × 10^−2^	1.24 × 10^−2^	2.47 × 10^−2^	3.19 × 10^−2^	3.68 × 10^−1^
PA1B3_HUMAN	1.67 × 10^−3^	8.50 × 10^−3^	8.81 × 10^−2^	2.05 × 10^−3^	4.29 × 10^−3^	2.07 × 10^−3^
AHNK2_HUMAN	3.16 × 10^−2^	5.67 × 10^−1^	6.50 × 10^−5^	1.38 × 10^−2^	1.08 × 10^−3^	1.08 × 10^−4^
AKAP2_HUMAN	8.66 × 10^−5^	3.41 × 10^−2^	2.08 × 10^−10^	1.22 × 10^−3^	8.42 × 10^−1^	2.13 × 10^−9^
RBM8A_HUMAN	3.47 × 10^−3^	1.64 × 10^−3^	4.38 × 10^−1^	1.64 × 10^−3^	4.92 × 10^−3^	1.64 × 10^−3^
HNRPQ_HUMAN	1.81 × 10^−3^	4.96 × 10^−2^	7.39 × 10^−1^	4.96 × 10^−2^	5.43 × 10^−3^	4.96 × 10^−2^
ICAM1_HUMAN	1.30 × 10^−4^	4.47 × 10^−2^	4.80 × 10^−3^	1.64 × 10^−3^	1.38 × 10^−3^	1.98 × 10^−1^
CRIP2_HUMAN	9.00 × 10^−1^	2.60 × 10^−2^	2.74 × 10^−10^	4.25 × 10^−2^	1.11 × 10^−3^	5.73 × 10^−8^
MVP_HUMAN	3.01 × 10^−5^	2.30 × 10^−2^	2.30 × 10^−2^	3.94 × 10^−3^	1.13 × 10^−2^	8.38 × 10^−1^
PRAF3_HUMAN	3.94 × 10^−3^	2.54 × 10^−2^	1.64 × 10^−7^	1.57 × 10^−1^	3.94 × 10^−3^	1.38 × 10^−3^
KAP2_HUMAN	8.66 × 10^−5^	7.07 × 10^−4^	6.96 × 10^−8^	8.13 × 10^−4^	8.42 × 10^−1^	1.50 × 10^−5^
LEG3_HUMAN	1.30 × 10^−4^	9.65 × 10^−3^	1.96 × 10^−4^	4.32 × 10^−3^	4.42 × 10^−1^	9.03 × 10^−3^
1433S_HUMAN	8.34 × 10^−2^	2.26 × 10^−2^	5.41 × 10^−5^	5.03 × 10^−4^	2.42 × 10^−7^	8.66 × 10^−5^
DEK_HUMAN	1.36 × 10^−3^	1.30 × 10^−4^	2.72 × 10^−1^	1.36 × 10^−3^	1.36 × 10^−3^	1.36 × 10^−3^
RANG_HUMAN	8.34 × 10^−4^	3.50 × 10^−11^	2.91 × 10^−1^	2.47 × 10^−2^	1.18 × 10^−9^	3.71 × 10^−9^
MFAP4_HUMAN	1.30 × 10^−4^	1.30 × 10^−4^	1.63 × 10^−2^	7.96 × 10^−1^	9.76 × 10^−4^	1.30 × 10^−4^
SRSF5_HUMAN	1.39 × 10^−3^	2.37 × 10^−2^	2.91 × 10^−1^	4.31 × 10^−2^	1.39 × 10^−3^	1.36 × 10^−3^
AT2A3_HUMAN	1.67 × 10^−3^	2.27 × 10^−2^	2.91 × 10^−1^	2.18 × 10^−2^	1.67 × 10^−3^	2.18 × 10^−2^
SC23A_HUMAN	2.36 × 10^−2^	7.73 × 10^−1^	1.44 × 10^−6^	5.78 × 10^−3^	2.36 × 10^−2^	1.37 × 10^−7^

**Table 4:** Excerption of the dysregulated biological pathways along with Normalized Enrichment Scores (NES).

Name	AC	SCLC	SqCC	LCC
KEGG_COMPLEMENT_AND_COAGULATION_CASCADES	−2.03	−1.98	−3.05	−1.54
KEGG_FOCAL_ADHESION	−2.51	−2.36	−2.46	−1.72
GOCC_ACTIN_CYTOSKELETON	NA	−2.05	−1.44	−1.65
GOCC_COLLAGEN_CONTAINING_EXTRACELLULAR_MATRIX	−2.17	−2.5	−2.84	−1.7
GOMF_CALCIUM_ION_BINDING	NA	−2.31	NA	NA
GOBP_ALTERNATIVE_MRNA_SPLICING_VIA_SPLICEOSOME	NA	2.58	NA	NA
GOBP_BIOLOGICAL_ADHESION	NA	−2.39	−1.68	NA
GOBP_CELLULAR_MACROMOLECULE_BIOSYNTHETIC_PROCESS	1.69	2.96	1.95	1.86
GOBP_CYTOPLASMIC_TRANSLATION	1.73	2.4	1.87	1.91
GOBP_ENDOCYTOSIS	NA	−2.39	−2.71	−1.65
GOBP_G_PROTEIN_COUPLED_RECEPTOR_SIGNALING_PATHWAY	NA	−1.81	−2.22	NA
GOBP_GENE_SILENCING_BY_RNA	1.64	2.45	1.72	1.35
GOBP_GLYCEROLIPID_METABOLIC_PROCESS	−2.15	−1.69	−1.85	NA
GOBP_HEMOSTASIS	−1.83	−1.86	−2.18	NA
GOBP_HUMORAL_IMMUNE_RESPONSE	NA	−1.85	−2.44	NA
GOBP_LYMPHOCYTE_MEDIATED_IMMUNITY	NA	−1.99	−2.17	NA
GOBP_MRNA_METABOLIC_PROCESS	1.61	3.49	1.78	1.33
GOBP_NCRNA_METABOLIC_PROCESS	1.63	2.42	1.9	2.11
GOBP_PEPTIDE_BIOSYNTHETIC_PROCESS	1.81	2.83	1.89	1.84
GOBP_PYRUVATE_METABOLIC_PROCESS	1.75	NA	1.55	1.43
GOBP_REGULATION_OF_CHROMOSOME_ORGANIZATION	NA	2.74	1.58	1.66
GOBP_REGULATION_OF_VESICLE_MEDIATED_TRANSPORT	NA	−1.97	−2.13	NA
GOBP_RHO_PROTEIN_SIGNAL_TRANSDUCTION	NA	−1.45	−2.13	NA
GOBP_RIBONUCLEOPROTEIN_COMPLEX_BIOGENESIS	1.79	2.88	2.05	1.94

## Data Availability

Experimental data has been submitted to the MassIVE data repository (https://massive.ucsd.edu/ProteoSAFe/static/massive.jsp, accessed on 22 April 2022) with the ID: MSV000089291.

## Supplementary Materials

The following supporting information can be downloaded at: https://www.mdpi.com/article/10.3390/cancers14112629/s1, Table S1: Detailed information about the samples included in the study; Table S2: Parameters of the software used, Table S3: List of proteins differentially expressed in all 4 subtypes of LC. Table S4: List of proteins differentially expressed only in one type of LC compared to tumor-adjacent tissue, Table S5: List of the 571 proteins with altered expression levels between the different types of LC, Table S6: Effect sizes of differences between tumorous and adjacent regions calculated for all proteins for LC types separately. Table S7: Results of the GSEA analysis.
